# The Role of Vitamin D in the Aging Adult

**DOI:** 10.12974/2309-6128.2014.02.02.1

**Published:** 2014-12

**Authors:** Meghan Meehan, Sue Penckofer

**Affiliations:** School of Nursing, Loyola University Chicago, IL., USA

**Keywords:** Aging, older adult, vitamin D

## Abstract

The number of individuals aged 65 and older is expected to more than double from 2012 to 2060. The role of vitamin D in the prevention and treatment of diseases associated with aging has not been well studied. Traditionally, the role of vitamin D focused on the maintenance of skeletal health in the older adult. With the discovery of vitamin D receptors in the nervous, cardiovascular and endocrine systems, the role of vitamin D and its impact on these systems has become an important area of research. Older adults are at risk for lower levels of vitamin D as a result of decreased cutaneous synthesis and dietary intake of vitamin D. Epidemiologic evidence indicates an association between low levels of vitamin D and diseases associated with aging such as cognitive decline, depression, osteoporosis, cardiovascular disease, hypertension, type 2 diabetes, and cancer. Clinical trials to determine the benefit of vitamin D supplementation in preventing and treating such diseases are in progress. This paper highlights current evidence regarding the role that vitamin D may play in diseases associated with aging and addresses the need for well-designed randomized trials to examine its benefit on health outcomes in the older adult.

## Introduction

Vitamin D plays a vital role in human health [35]. Low levels of vitamin D can drastically impact a person's physical and mental well-being [22, 36]. Traditionally, research focused on the role of vitamin D in the maintenance of skeletal health. In recent years, following the discovery of vitamin D receptors throughout the body, its role in the prevention and treatment of chronic diseases has become an important area of study. Vitamin D deficiency has been linked to various health problems, including cognitive decline, depression, osteoporosis, cardiovascular disease, hypertension, diabetes, and cancer [36, 39]. As persons age, the risk for vitamin D deficiency significantly increases. The percent of older adults suffering from vitamin D deficiency ranges from 20 to 100% in the United States (US)[37]. Risk factors contributing to vitamin D deficiency in older adults include reduced nutritional intake of vitamin D, increasing adiposity, decreased cutaneous synthesis of vitamin D, and less time spent outdoors (Table 1) [22, 21]. The number of individuals aged 65 and older is expected to more than double from 2012 to 2060 [67]. Thus, understanding the relationship between vitamin D and chronic diseases in the older adult and whether treatment of vitamin D deficiency can prevent or ameliorate these disorders is important. This paper highlights the current evidence regarding the role that vitamin D may play in diseases associated with aging and also addresses the need for randomized clinical trials (RCTs) examining the impact of vitamin D on the prevention and treatment of these diseases in the older adult.

## Pathophysiology

Vitamin D can be obtained through diet, supplements, and sunlight. Vitamin D is naturally present in oily fish such as cod liver oil, swordfish, and salmon (vitamin D_3_) and ultraviolet (UV) exposed mushrooms (vitamin D_2_), fortified in products such as milk and orange juice, and available as vitamin D_2_ and D_3_ supplements [39]. Exposure to solar UV radiation causes the conversion of 7-dehydrocholesterol in the skin to form pre-vitamin D_3_ and later vitamin D_3_. Vitamin D_2_ and D_3_ from dietary ingestion and skin circulates first to the liver where it undergoes hydroxylation by vitamin D 25-hydroxylase (25-OHase) to 25 hydroxyvitamin D [25(OH)D_3_]. [25(OH)D_3_] then circulates to the kidneys where it undergoes a second hydroxylation by 25-hydroxyvitamin D-1-α-hydroxylase (1-OHase) to 1,25-dihydroxyvitamin D [1,25(OH)_2_D], the biologically active form of vitamin D (Calcitriol). As the concentration of [1,25(OH)_2_D] increases, vitamin D nuclear receptors (VDR) throughout the body become stimulated, causing activation of gene transcription. Interaction between [1,25(OH)_2_D] and VDR sites in various organs of the body produce numerous biological actions affecting the potential for the development of many diseases. These biological actions include, regulation of calcium and phosphorus in the intestines and bones (osteoporosis), insulin sensitivity and secretion (diabetes), regulation of cellular growth and angiogenesis (immune regulation and cancer), renin expression and inhibition of vascular smooth muscle proliferation (hypertension and cardiovascular disease), and inflammation and amyloid plaque formation in the brain (cognitive decline and Alzheimer's disease) [39].

### Vitamin D Recommendations

Vitamin D supplementation is the most appropriate treatment option for the older adult population [22]. Without adequate exposure to sunlight, it is almost impossible to achieve sufficient levels of vitamin D from nutritional sources and, thus, supplementation has been suggested by numerous experts as a safe and cost-effective alternative to treating vitamin D deficiency [37]. Recently, the Institute of Medicine (IOM) published new dietary guidelines regarding vitamin D supplementation. According to the report, children older than 1 years old and adults up to 70 years of age are recommended to consume 600 IU of vitamin D daily and adults greater than 70 years of age are recommended to consume 800 IU of vitamin D daily [40].

### Side Effects of Taking Vitamin D

Although vitamin D deficiency is commonly seen in practice, vitamin D toxicity is very rare [21]. Vitamin D toxicity is defined by most experts as having a serum 25-hydroxyvitamin D [25(OH)D] concentration of more than 150 ng/mL [55]. Vitamin D toxicity is caused by excessive ingestion of vitamin D supplements over an extended period of time and is not attributed to prolonged sunlight exposure [21]. Clinical signs and symptoms of vitamin D toxicity are often vague, such as poor appetite, weight loss, increased urination, and arrhythmias [55]. Additionally, high levels of vitamin D for long periods have been attributed to hypercalcemia and hyperphosphatemia and, subsequently, cardiovascular and kidney damage [55]. Therefore, patients who are being treated for vitamin D deficiency with high doses of vitamin D supplementation for a long period of time should have their lab values monitored closely [21].

### Psychological Aging

Evidence suggests that vitamin D plays a crucial role in brain development and cognitive performance [22]. Several vitamin D receptors have been identified in regions of the brain that affect cognition and mood, suggesting that low vitamin D levels may be associated with cognitive decline and symptoms of depression [43, 34].

### Cognitive Decline

Cognitive decline is a devastating mental health problem that includes a spectrum of illnesses, ranging from mild cognitive impairment to advanced stages of dementia. As a person ages, their risk for cognitive decline increases dramatically; affecting nearly 25% of all persons 65 and older in the US [19]. Recent findings suggest that low vitamin D levels in older adults are associated with an increased incidence of cognitive decline [49].

Four cross-sectional studies, two from Europe and two from the US, examined the relationship between vitamin D and cognitive decline. One study of 1766 individuals aged 65 and older from England, reported that those with low serum 25(OH)D levels had an increased risk for developing cognitive impairment [50]. Another study of 3,133 European men, aged 40 to 79, revealed that older men with low levels of serum 25(OH)D (<35 nmol/l), had reduced speed in processing information [46]. Llewellyn and colleagues [48] examined 3,325 persons aged 65 and older and reported that individuals with low levels of vitamin D (<25 nmol/l) had a four times greater chance of developing cognitive impairment than individuals with higher vitamin D levels (>75 nmol/l). Contrary, a subsequent study of 1080 US adults aged 65 and older reported that low vitamin D levels (< 20 ng/mL) had no effect on a person's memory, but rather, had a significant negative effect on a person's ability to perform executive functions, focus their attention, and process information [15]. Although these cross-sectional studies reveal promising data regarding vitamin D and cognition, the studies varied in how they defined vitamin D deficiency. Additionally, the associative findings from the cross-sectional designs may be due to the aging process itself rather than low vitamin D levels.

A prospective study conducted by Llewellyn and colleagues [49], followed 858 Italian individuals, aged 65 and older for over six years and found that older adults with severely low levels of 25(OH)D (<25 nmol/L) were at an increased risk of developing significant cognitive decline. However, a prospective study conducted in the US followed 1,604 elderly men, aged 65 and older for almost five years, and found no significant relationship between vitamin D levels and cognitive function or decline [62]. Although 179 men had cognitive impairment at baseline and over 200 men developed cognitive impairment during the study, the association between low vitamins D levels and cognitive decline was not significant after adjusting for covariates, such as race and educational status.

A recent prospective cohort study examined the relationship between vitamin D levels and cognitive performance in 2,777 older adults, aged 70 to 79, who participated in the Health, Aging, and Body Composition Study (Health ABC study) in the US [72]. Sixty-eight percent of individuals had vitamin D levels less than 30 ng/ml. Findings indicated that lower vitamin D levels were associated with lower baseline scores on the Mini-Mental State Examination as well as a greater decline in cognition over four years when compared to those with higher vitamin D levels. However, further clinical trials are needed to determine whether vitamin D supplementation can reduce cognitive decline. In the Women's Health Initiative, a sub-study on memory was conducted to examine the benefit of vitamin D and calcium supplementation on cognitive impairment [57]. No significant findings emerged; however, this could be attributed to the few cases of incident cognitive impairment during the course of the trial and the low daily dose of vitamin D (400 IUs). The dose of 400 IUs is not congruent with the Institute of Medicine (IOM) [40] daily recommended amount for adults; which is 600 (ages 1 to 70) to 800 IUs (> 70 years).

Therefore, prospective studies and RCTs with larger, diverse samples are needed to confirm suggested associations between low vitamin D levels and cognitive decline and to determine whether vitamin D supplementation is an efficacious intervention for minimizing and/or preventing cognitive impairment [27]. In addition to cognitive impairment, Alzheimer and vascular dementia are now being examined relative to vitamin D in the elderly. This year, a large, prospective, population-based study of 1658 ambulatory, older adults (mean age 74) who participated in the United States Cardiovascular Health Study were examined for the relationship between low vitamin D levels and increased risk of all-cause dementia, including Alzheimer disease [47]. Over the course of 5.6 years, 171 cases of all-cause dementia and 102 of Alzheimer disease were found. Researchers reported more than double the risk for both all-cause dementia (Hazard Ratio: 2.25, 95% CI: 1.23-4.13) and Alzheimer disease (Hazard Ratio: 2.22, CI: 1.02-4.83) for older adults with severe vitamin D deficiency (<25nmol/L). Schlogl and Holick [60] recently summarized new clinical data suggesting associations between low vitamin D and these disorders. They emphasized the need for well-designed clinical trials to assess the benefits of vitamin D in persons with varying neurocognitive deficits. Currently, the VITamin D and OmegA-3 Trial (VITAL) is conducting an ancillary RCT examining the benefit of vitamin D and/or omega-3 on memory loss and cognitive decline in over 25,000 adults (both men aged 50 and women aged 55 and older) who are receiving supplements or placebo over a period of five years (http://www.vitalstudy.org/Studies.html).

### Depression

Depression is a disabling mental health problem that negatively impacts a person's thoughts, actions, and feelings [2]. Depression is commonly experienced by older adults and occurs more frequently in women than men [2]. Recently, it was reported that in older primary care patients (mean age 73.8 years) about one-fifth have vitamin D deficiency. Additionally, those with severe deficiency were more likely to be older, frail, and have more frequent depression [44].

Early work by Hoogendijk and colleagues [38] found, in a cohort study of over 1,200 persons aged 65 and older, that levels of 25(OH)D were 14% lower in persons with minor depression and 14% lower in persons with major depressive disorder when compared to controls. The use of a diagnostic assessment of depression was strength of this study. One cross-sectional study examined 2,070 individuals, aged 65 and older, who had completed the 2005 Health Survey for England and provided blood specimens [64]. Findings indicated that depressive symptoms were significantly associated with individuals who had vitamin D levels less than 10 ng/mL. A limitation of this study was the self-report measure of depression. Another cross-sectional study followed 12,594 adults, aged 20 to 90, from the Cooper Clinic over a period of four years [34]. They found a significant correlation between low vitamin D levels and symptoms of depression, particularly in persons with a history of clinical depression. Although the study was large, there was a lack of racial and ethnic diversity. Findings from the Women's Health Initiative Observational Study of postmenopausal females, aged 50 to 79, who were followed for over three years, revealed an inverse relationship between vitamin D from dietary sources and symptoms of depression [9]. The study found that women with diets rich in vitamin D had a 20% lower risk of developing depression. Although women who consumed between 100 and 800 IUs of vitamin D supplementation had a decreased risk of developing depression, the results revealed no association between consumption of vitamin D supplementation and depressive symptoms. A limitation of this study was the self-report measure of depression.

Some studies have examined the relationship between depression and taking vitamin D supplements. A RCT conducted by Sanders and colleagues [58] investigated the effects of a yearly, large dose of 500,000 IU of vitamin D3 on depressive symptoms in 2,258 women, aged 70 and older, over the course of three to five years. The results, from a subgroup of 150 women whose blood was analyzed, concluded that although serum 25(OH)D levels were 41% higher in the women who received the vitamin D supplements versus the placebo after one year, no significant change in mood or mental well-being was found. Similar results were reported by Kjaergaard and colleagues [43] who examined the effects of a weekly, large dose of 40,000 IU of vitamin D3 on depressive symptoms in 243 adults, aged 30 to 75, over the course of six months. The findings revealed that low vitamin D levels were associated with symptoms of depression; however, vitamin D supplementation showed no improvement in depressive symptoms. Limitations of the study were the short time frame, self-report of depressive symptoms by participants, and the exclusion of individuals who were diagnosed with depression or who took anti-depressants.

A recent systematic review and meta-analysis of vitamin D deficiency and depression in adults included one case-control study, ten cross-sectional studies and three cohort studies for the analysis [3]. Findings revealed lower levels of vitamin D in persons with depression compared to controls, especially when comparing individuals with the lowest to the highest vitamin D categories. The authors recommended the need for RCTs to determine the effect of vitamin D in the prevention and treatment of depression.

### Physical Aging

Current evidence suggests that low vitamin D levels may contribute to the development of diseases of aging, such as osteoporosis, cardiovascular disease, hypertension, type 2 diabetes, and cancer [22]. Consequently, numerous research studies are currently exploring the efficacy of vitamin D supplementation in preventing and treating common co-morbidities in the older adult population.

### Osteoporosis

Approximately 10 million adults, over the age of 50, suffer from osteoporosis and 34 million have reduced bone mass, or osteopenia [53]. Although osteoporosis affects all sexes, races, and ethnicities, 80% of individuals with osteoporosis are women and 20% are Caucasian [53]. Numerous factors increase a person's risk for osteoporosis, including inadequate consumption of calcium and vitamin D, inactivity, tobacco use, age over 50, female gender, menopausal status, heredity, and thin body frame [53].

Evidence has confirmed that vitamin D and calcium play a vital role in supporting the health of the skeletal system [40]. Low levels of vitamin D cause reduced calcium absorption in the intestines, leading to increased parathyroid hormone levels and increased bone turnover and, subsequently, osteopenia and osteoporosis [69]. Research has suggested a positive correlation between low vitamin D levels and increased risk of falls and fractures, muscular weakness, and poor physical functioning and balance; however, findings are inconsistent [40, 24].

Several meta-analysis and systematic reviews have examined the effect of vitamin D supplementation on fracture and fall risk. One early meta-analysis of 20 large RCTs examined non-vertebral and hip fractures in over 83,000 adults greater than 65 years of age [11]. Findings revealed that non-vertebral and hip fractures were reduced by almost 20% in individuals who consumed greater than 400 IU of vitamin D supplementation each day [11]. A more recent pooled analysis of 11 RCTs by the same investigators found that higher doses of vitamin D (>800 IUs/day) helped to prevent hip and non-vertebral fractures in individuals greater than 65 years of age [10].

A Cochrane systematic review examined 45 RCTs of vitamin D and vitamin D analogues for preventing fractures in over 84,000 older adults [8]. It was found that vitamin D supplementation was not effective in preventing hip or vertebral fractures; however, administration of calcium and vitamin D together, was helpful in decreasing hip and non-vertebral fractures in frail elderly who were institutionalized. Similar results were found in an analysis of over 68,000 adults from 7 RCTs from both the US and Europe [28]. Participants who received vitamin D and calcium supplementation had a significantly decreased risk of all fractures, whereas participants who only received vitamin D supplementation showed no significant change in fracture risk.

Another review examined the past 15 years of literature on vitamin D and falls and found that vitamin D supplementation of at least 800 IUs effectively decreased the risk of falls in the older adult population [6]. Additionally, vitamin D was also associated with improved balance, gait, and physical functioning and, in turn, reduced incidence of falls. Conversely, a RCT conducted by Sanders and colleagues [59] reported an increased incidence of falls in older persons taking vitamin D. The study examined 2,258 women over age 70 that were living within the community and were given a yearly dose of 500,000 IU of vitamin D supplementation for three to five years. Findings revealed that 74% of women who received the vitamin D supplement and 68% of women who received the placebo fell at least once during the study. In addition, 171 women who received the vitamin D supplement and 125 women who received the placebo sustained a fracture during the study. It was suggested that the increased falls and subsequent fractures in the individuals who consumed the large dose of vitamin D supplementation may be due to enhanced muscle tone and strength and improved mood which lead to increased mobility and, consequently, a greater risk for falls [26].

Most recently, a meta-analysis of elderly fallers and non-fallers has addressed concerns raised by previous research. For this analysis, 18 observational studies (10 cross-sectional and 8 cohort studies) were re-examined to determine the difference in risk of falling according to vitamin D serum concentrations (25-OHD) [4]. They reported that fallers have a notably lower level of vitamin D (< 20 ng/ml) than non-fallers. The US Preventive Services Task Force recommends that exercise, physical therapy and/or vitamin D supplementation be used to prevent falls for community dwelling older adults aged 65 and older who are at risk for falls [52]. Finally, the American Geriatric Society Workgroup on vitamin D supplementation for older adults [1] recommends that a serum vitamin D level of 30ng/mL be a minimum goal to achieve in this group, particularly for those who are frail, at risk for falls, injuries or fractures. They reported that unless there was risk for hypercalcemia (advanced renal disease, certain malignancies, or sarcoidosis), then there is no risk in taking 1000 IUs per day.

Thus, it is evident that vitamin D is an essential nutrient needed for skeletal health. Vitamin D supplementation appears to be a promising solution to reduce fall and fracture risk among the older adult population; however, future research is still needed particularly in the area of supplement dosing.

### Cardiovascular Disease

Cardiovascular disease is the primary cause of mortality in the world, with approximately 17 million individuals dying worldwide annually [75]. The role of vitamin D and its impact on the cardiovascular system gained interest a number of years ago due to epidemiologic evidence suggesting its beneficial effect on heart disease [70]. More contemporary work has called to question the efficacy of vitamin D on cardiovascular events.

A systematic review conducted by Wang and colleagues [71] analyzed 17 prospective studies and RCTs that evaluated vitamin D and calcium supplementation and cardiovascular events. The researchers found that individuals who consumed moderate to large amounts of vitamin D supplementation, approximately 1000 IU of vitamin D daily, had a slight, but not significant decrease in cardiovascular risk, whereas calcium supplementation showed no evidence of cardiovascular risk reduction [71]. A systematic review and meta-analysis performed by Elamin and colleagues [29] examined 51 RCTs that investigated the effects of vitamin D supplementation on cardiovascular outcomes. The results showed no significant relationship between vitamin D supplementation and reduction in cardiovascular mortality and risk for heart attack and stroke. The results from the analysis are limited; however, due to the fact that most of the studies were not designed to examine cardiovascular outcomes and many of the studies had a small sample size. Sun and colleagues [65] examined the relationship between vitamin D levels and stroke risk using data from the Nurses' Health Study (n=32,826) and compared the results with six prospective studies. Findings indicated that low levels of vitamin D were modestly correlated with a risk for stroke in women who had no prior history of a stroke. A limitation of this report was that only female nurses were represented and the sample was primarily Caucasian.

Although numerous studies propose a potential inverse relationship between vitamin D and cardiovascular disease [41, 65], evidence from large RCTs is limited. A recent Cochrane review on vitamin D supplementation for prevention of mortality in adults [12] generated data from 56 RCTs that used vitamin D supplementation (cholecalciferol, ergocalciferol, alfacalcidol, calcitriol) for an average of over four years. Findings indicated that vitamin D_3_ seems to decrease overall mortality in the elderly who were not dependent on help or living in institutional care. However, no significant effect on cardiovascular mortality was reported for all participants. Similarly, a meta-analysis of observational cohort and randomized intervention studies reported comparable findings in that vitamin D_3_ supplementation reduced overall mortality among older adults; however, the researchers recommend the need for the future study of optimal dosing and duration [23].

Currently, there are two ongoing large RCTs examining the effects of vitamin D supplementation on cardiovascular disease, the ViDA study, or Vitamin D Assessment, in New Zealand, and the VITAL study, or Vitamin D and Omega-3 Trial, in the US [18]. Unfortunately, the results from these studies are not currently available. Consequently, because the current evidence is inconsistent, conclusions regarding vitamin D supplementation and cardiovascular disease cannot be drawn at this time.

### Hypertension

Hypertension is a major risk factor contributing to cardiovascular disease [74]. The risk for hypertension increases significantly as a person ages. Therefore, early detection and treatment of high blood pressure is of utmost importance in older adults [74]. Several cross-sectional and prospective studies have proposed an association between vitamin D and high blood pressure [14]. One meta-analysis which examined 18 studies, 4 prospective and 14 cross-sectional, reported an inverse relationship between vitamin D levels and hypertension and found that for every 40 nmol/l increase in vitamin D level, the risk for hypertension was reduced by 16% [17]. Observational data from a number of systematic reviews has also demonstrated an association between low vitamin D levels and an increased incidence of high blood pressure as well as risk for hypertension [66, 68, 56]. RCTs from these same reviews; however, conflict with the observational data and fail to confirm a relationship between vitamin D supplementation and reduction in blood pressure [66, 68, 56]. Variability in sample size, type of population, length of study, and vitamin D dosages have been reported as possible reasons for the inconsistency in the data findings.

A recent RCT of persons aged 70 and older with isolated systolic hypertension and vitamin D levels less than 30 ng/mL found no improvement in blood pressure following supplementation [73]. Participants received either 100,000 IUs of cholecalciferol or a matching placebo every three months for one year. A major study limitation was that although vitamin D levels increased from 18 ng/ml to 28 ng/ml, this may not have been sufficient to alter blood pressure [73]. Therefore, although a relationship between vitamin D and hypertension has been reported, clinical trials are needed to determine the impact vitamin D supplementation has in treating and/or preventing hypertension before recommendations can be made [68].

### Type 2 Diabetes

The incidence of diabetes in older adults is alarming with almost 11 million or 30% of the population over the age of 65 affected by diabetes in 2010 [20]. Major complications of diabetes include cardiovascular disease, hypertension, and stroke [20]. Therefore, measures to combat the incidence of diabetes and reduce the occurrence of diabetes-related co-morbidities are of utmost importance.

Accumulating evidence suggests that a disruption in vitamin D regulation within the body may contribute to the development of type 2 diabetes [30]. For this reason, correction of vitamin D status with supplementation has been proposed as a potential solution to help prevent and treat diabetes. Although several cross-sectional and prospective studies report an association between low vitamin D levels and an increased risk for diabetes, findings are inconsistent [32, 42]. One cross-sectional study evaluated levels of vitamin D and blood sugar in 2,038 adults from England who were greater than age 65 and also completed a health questionnaire [33]. They concluded that high levels of blood sugar were independently correlated with vitamin D levels less than 25 nmol/L.

Several RCTs have examined the benefit of vitamin D supplementation on diabetes outcomes. Two RCTs have reported conflicting results on the efficacy of vitamin D supplementation on improving insulin secretion. In one RCT, 92 obese adults consumed 2,000 IU of vitamin D once a day or 400mg of calcium twice a day for 16 weeks [51]. They found a significant improvement in beta cell functioning and insulin secretion in participants who took the vitamin D supplement, but not in those who only consumed the calcium supplement. Another RCT reported contrary findings [25]. In adults with pre-diabetes (hemoglobin A1C values between 5.8 and 6.9 %) and baseline vitamin D levels less than 30 ng/mL, following a mean weekly dose of 88,865 IUs of vitamin D (n=56), there was no change in insulin secretion, insulin sensitivity, or diabetes incidence compared to placebo (n=53) after one year. However, vitamin D supplementation increased vitamin D levels from an average of 22 to 70 ng/mL and slightly reduced hemoglobin A1C levels by 0.2%. Several systematic reviews support these findings. One review analyzed 15 RCTs and found that vitamin D supplementation did not improve fasting blood sugar, hemoglobin A1C, or insulin resistance in persons without diabetes. However, in those individuals who were diabetic or pre-diabetic, a slight improvement in fasting blood sugar and insulin resistance was found [31]. Another review examined 12 cross-sectional and 10 RCTs and found data to be inconsistent regarding insulin sensitivity and secretion [32]. This may be due to the short duration of supplementation (less than six months) and/or varying doses of supplements (400 IUs to 120,000 IUs). At this time, there is insufficient evidence to determine whether low levels of vitamin D contribute to the development of type 2 diabetes. Therefore, large RCTS with beneficial dosing of vitamin D are needed before supplementation can be recommended as an effective preventative therapy for diabetes management. One RCT currently in progress is the D2D study (http://www.d2dstudy.org/) which will determine whether 4000 IUs daily or a placebo taken over a period of four years will modify diabetes risk in persons at risk for diabetes.

### Immune Function

The immune system is our body's defense against harmful bacteria, viruses, parasites, fungus, and cancers [16]. As a person ages, their immune system gradually deteriorates. This natural process is known as immunosenescence. The immune system of an older adult progressively loses its ability to fight infections and develop active immunity following vaccinations, leading to an increase in elder mortality rates [16]. Vitamin D helps to regulate the innate and adaptive immune responses through T-cell differentiation and expression of cathelicidin, a protein that kills infectious organisms, like tuberculosis bacillus [39, 7].

### Cancer

Epidemiologic evidence has suggested that higher levels of vitamin D may reduce the risk of developing several cancers; most commonly, breast, prostate, and colon. Researchers have suggested that the transformation of 25(OH)D to 1,25(OH)_2_D in healthy cells of the breast, prostate, and colon may help to prevent cancer by stimulating cellular maturation and apoptosis, inhibiting angiogenesis and oxidative stress, and expressing genes to control cellular proliferation [39].

A reanalysis of the 36,282 post-menopausal females from the Women's Health Initiative found that 15,646 (43%) women who were not taking personal calcium or vitamin D supplements prior to study randomization, had a significant risk reduction in breast cancer by 14 to 20% and a non-significant risk reduction in colorectal cancer by 17% [13]. A meta-analysis of 5372 post-menopausal women found that the dosage of vitamin D supplementation did not affect breast cancer risk; however, the combination of vitamin D and calcium showed a slight, non-significant reduction of breast cancer risk [63]. In a prospective study of 1260 men with prostate cancer and 1331 control subjects, a 57% risk reduction of lethal prostate cancer was noted in individuals with higher 25(OH)D levels; however, there was no statistically significant correlation between 25(OH)D levels and non-lethal prostate cancer [61]. A meta-analysis of 8 prospective studies and the Physicians' Health Study including 1,822 colon and 868 rectal cancers, found a significant inverse relationship between 25(OH)D levels and colorectal cancer, especially for rectal cancer [45]. Although numerous studies suggest the use of vitamin D supplementation in cancer risk reduction, further investigation of vitamin D dosing is needed through RCTs and meta-analysis. Future large RCTs such as the VITAL study are currently underway and should provide a wealth of knowledge regarding cancer and vitamin D.

## Conclusion

Vitamin D deficiency is a common, serious medical condition that significantly affects the health and well-being of older adults. Evidence has confirmed the association between vitamin D and osteoporosis; however, at this time, RCTs are needed to determine whether providing vitamin D can help to prevent, treat, or ameliorate the chronic conditions of aging such as cognitive decline, depression, cardiovascular disease, hypertension, type 2 diabetes, and cancer (Table 2). A comprehensive systematic review recently published reported similar findings. Although new studies have shown promising data regarding vitamin D's role in various health outcomes, such as bone and cardiovascular health, cancer, and the immune system, the findings are inconsistent and no firm conclusions can be drawn at this time [54]. As our nation's older adult population continues to grow, establishing universal guidelines for testing and treating vitamin D deficiency is needed. Although some have suggested the need to specify standard vitamin D values for certain disorders [5], much work in this area is warranted. Finally, research to examine the dosing of vitamin D supplements necessary to prevent the chronic diseases of aging would have significant benefit for future generations.

## Figures and Tables

**Figure 1:**
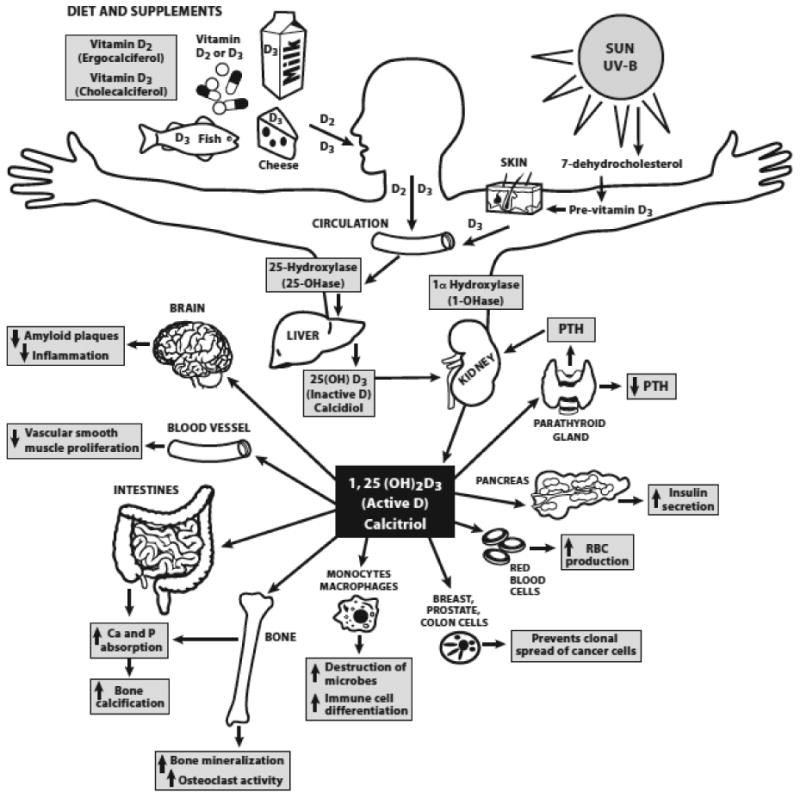
Physiology of Vitamin D.

**Table 1:** Factors Contributing to Vitamin D Deficiency/Insufficiency in the Aging Adult

Risk Factors
Age > 50
Female Gender
Dark Skin Pigmentation
Poor Skin Integrity
Reduced Time Spent Outdoors
Decreased Intake of Vitamin D
Obesity (BMI >30)
Malabsorption Disorders
Reduced Renal Function
Medications (e.g. Anticonvulsants)

**Table 2:** Vitamin D Deficiency and Diseases in the Aging Adult

**Cognitive Decline**
Associated with cognitive decline Associated with ↑ risk for dementia Associated with ↑ risk for Alzheimer disease Mechanisms may include inflammation and formation of amyloid plaque in the brain
**Depression**
Associated with major depression Associated with ↑ depressive symptoms Mechanisms may include neuroimmunomodulation and regulation of neurotrophic factors in the brain
**Osteoporosis**
Evidence for ↑ risk for low trauma fracture Associated with ↑ risk for falls Mechanisms include ↑ PTH and bone turnover
**Cardiovascular Disease**
Associated with ↑ risk for cardiovascular morbidity and mortality Associated with ↑ stroke in women Mechanisms may include smooth muscle proliferation and inflammatory processes
**Hypertension**
Associated with ↑ risk for hypertension Mechanisms may include alterations in the regulation of the renin-angiotensin system
**Type 2 Diabetes**
Associated with higher fasting glucose levels Associated with ↑ risk for insulin resistance Potential mechanisms may include insulin sensitivity and secretion
**Cancer**
Associated with ↑ risk for colorectal cancer, more specifically rectal cancer Associated with ↑ risk for metastatic prostate cancer Potential mechanisms may include alterations in the autoimmune response and cellular proliferation
